# ASO Author Reflections: Liquid Biopsy in Gastrointestinal Malignancies: Role of Exosomes

**DOI:** 10.1245/s10434-023-13270-4

**Published:** 2023-03-01

**Authors:** Amber Gonda, Maheswari Senthil

**Affiliations:** 1 Department of Cell Biology and Physiology, Brigham Young University, Provo, UT USA; 2 Division of Surgical Oncology, Department of Surgery, University of California at Irvine, Irvine Medical Center, Orange, CA USA

Liquid biopsies offer noninvasive, real-time access to the molecular status of various cancers. As the field of liquid biopsy expands and the scope of its clinical utilization broadens, novel tools that provide deeper insights into tumor biology and behavior are necessary. The most commonly performed liquid biopsy, for circulating tumor DNA (ctDNA) (tumor-informed and tumor agnostic), has been rapidly integrated in the management of gastrointestinal (GI) malignancies; however, it has serious limitations. ctDNA is released by necrotic and apoptotic cells through a passive process and its sensitivity is affected by tumor type, location, and vascularization. Our group recently reported that the plasma levels of ctDNA in GI peritoneal carcinomatosis (PC) are lower than other metastases and are unreliable to detect somatic gene alterations.^1^ Similar results have been reported by others in gastric and colon cancer.^2,3^ Hence there is an urgent need to develop alternate liquid biopsies to bridge this gap, particularly since ctDNA is being evaluated in clinical trials for deescalation of treatment in GI malignancies.

Exosomes, nanovesicles (30–160 nm) secreted in abundance by the cancer cells are packed with information that have been implicated in cancer progression, creation of premetastatic niche, epithelial–mesenchymal transition, and metastasis.^4^ Recent studies of blood and peritoneal fluid exosomes in GI malignancies have shown their diagnostic, prognostic, and predictive capabilities. Since the biogenesis, loading of information, and release of exosomes are highly regulated processes in live cells, the information carried in tumor-derived exosomes represent the status of actively metabolizing cancer cells and can provide deeper insights. Furthermore, evaluation of peripheral blood exosomes provides information about the overall outcome of the tumor and its influence on the rest of the host systems and is vitally important to understand the overall status of the patients’ condition.

The current study by our group is an initial exploratory study to evaluate exosome gene expression in patients with non-metastatic colon cancer, visceral metastasis, and PC.^5^ We found that exosomes extracted from peripheral blood carry distinct genetic signatures in colon cancer compared with healthy volunteers. The major significance of this study was that this signature, ExoSig445, was able to differentiate PC from healthy volunteers with 100% sensitivity, offering a clear signal for further investigation of this gene signature in PC. Interestingly, 58 of the highly differentially expressed genes are shared with the colon adenocarcinoma tissue gene expression reported in the Cancer Genome Atlas (TCGA). Several of these commonly shared genes have been associated with cancer metastasis and prognosis. The results of the current study are being validated in a prospective longitudinal study of patients with colon cancer to further refine and develop a tool that is clinically meaningful.

As with any nascent field, there are practical challenges in the translation of exosomal liquid biopsy into clinical use. There needs to be a concerted effort to standardize the techniques for isolation of exosomes from small volume biofluids, develop internal controls and reference genes that are reproducible, and create uniform standards for gene expression analysis. Nevertheless, advances in these areas are being made with new exosome diagnostic tests reaching the market, such as ExoDx Prostate Intelliscore (EPI) test, with breakthrough device designation approval by the FDA.^6^ In summary, the potential of exosomes as a liquid biopsy tool is unlimited and warrants serious consideration.
